# Consistent spatial patterns in microbial taxa of red squirrel gut microbiomes

**DOI:** 10.1111/1758-2229.13209

**Published:** 2023-11-09

**Authors:** Alicia Halhed, Lauren Petrullo, Stan Boutin, Ben Dantzer, Andrew McAdam, Martin Wu, Karl Cottenie

**Affiliations:** ^1^ Department of Integrative Biology University of Guelph Guelph Canada; ^2^ Department of Biology Carleton University Ottawa Canada; ^3^ Department of Psychology University of Michigan Ann Arbor Michigan USA; ^4^ Department of Ecology & Evolutionary Biology University of Michigan Ann Arbor Michigan USA; ^5^ Department of Biological Sciences University of Alberta Edmonton Canada; ^6^ Department of Ecology and Evolutionary Biology University of Colorado Boulder Colorado USA; ^7^ Department of Biology University of Virginia Charlottesville Virginia USA

## Abstract

Gut microbiomes are diverse ecosystems whose drivers of variation remain largely unknown, especially in time and space. We analysed a dataset with over 900 red squirrel (*Tamiasciurus hudsonicus*) gut microbiome samples to identify the drivers of gut microbiome composition in this territorial rodent. The large‐scale spatiotemporal replication in the data analysed was an essential component of understanding the assembly of these microbial communities. We identified that the spatial location of the sampled squirrels in their local environment is a key contributor to gut microbial community composition. The non‐core gut microbiome (present in less than 75% of gut microbiome samples) had highly localised spatial patterns throughout different seasons and different study areas in the host squirrel population. The core gut microbiome, on the other hand, showed some spatial patterns, though fewer than in the non‐core gut microbiome. Environmental transmission of microbiota is the likely contributor to the spatiotemporal distribution observed in the North American red squirrel gut microbiome.

## Graphical abstract

Cycle of environmental transmission leading to microbial colonization of North American red squirrel gut microbiomes.

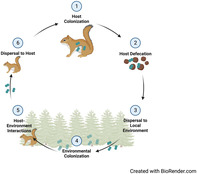

## INTRODUCTION

Microbiomes are dynamic communities that are increasingly accessible for exploratory and manipulative research as modern metagenomic and bioinformatics tools become more robust (Amato et al., 2019; Goodrich et al., 2014; Ma et al., 2014; Miller et al., 2018; Xia & Sun, 2017). Microbiome sampling provides valuable information on the community dynamics of microbiota within an ecosystem when analysed in conjunction with ecological data from well‐studied plant and animal populations. These microbial communities are diverse and important to their surrounding ecosystem, which may include improving ecosystem health by maintaining the integrity of nutrient cycles or impacting host response to disease pressure.

The core microbiome is generally defined based on the consistent detection (high occupancy) of a microbial taxon in many (30%–95%+) of the microbiome samples from a given sampling site (Risely, 2020). In addition to being consistently detected, a microbial taxon designated as core should also be relatively abundant in individual microbiomes. Specific core definitions vary between 1% and 6% relative abundance (Hernandez‐Agreda et al., 2017; Risely, 2020; Shade & Stopnisek, 2019). Other definitions of a core microbiome exist outside this definition, considering factors such as microbial function or temporal changes in community composition (Risely, 2020). While less abundant and/or lower occupancy, the non‐core microbiome may also provide important functions for the success of its host (Compant et al., 2019; Hol et al., 2015; Risely, 2020). These non‐core microorganisms can be strongly influenced by host location (Shaw et al., 2017), suggesting a key role of the host environment in microbiome assembly.

North American red squirrels (*Tamiasciurus hudsonicus*) defend exclusive individual territories, which implies discrete space use of the approximately 3400 m^2^ that a red squirrel will occupy on average (Lamontagne et al., 2013). Nevertheless, territorial intrusions on neighbouring territories do occur, likely to pilfer hoarded food from neighbours (Donald & Boutin, 2011). The act of pilfering may result in indirect transmission of microbial taxa between squirrels via the stolen food item. While the origin of microbial taxa on a food item is uncertain (e.g., from a squirrel or their environment), stored food items develop unique microbial profiles based on their storage locations (Ding et al., 2015). When those food items are eventually consumed, the food microbiome may colonise the oral and/or gut microbiomes of the new host (Adler et al., 2016; Schmidt et al., 2019). Meanwhile, direct transmission of microbial taxa between squirrels may also occur through brief physical interactions during mating. Physical interactions are quite rare when a squirrel attempts to defend their territory (Dantzer et al., 2012). While direct interactions are a potential transmission of microbial taxa between hosts during the mating season, localised patterns in gut microbial community composition can be expected largely due to indirect transmission.

The Kluane Red Squirrel Project (KRSP) is a long‐term study of a population of North American red squirrels living near Kluane National Park in Yukon, Canada (Dantzer et al., 2020). KRSP researchers collected over 900 faecal microbiome samples from 2008 to 2010 (Ren et al., 2017). This large sample size and temporal replication make these data unique for examining spatial effects on microbial communities (Ramette et al., 2009). The original analysis of these data identified that external ecological factors were key drivers of gut microbiome composition (Ren et al., 2017). While Ren et al. (2017) tested large‐scale spatial patterns at the 0.2–7.3 km scale across seasons, we hypothesised that small‐scale spatial patterns at less than 1 km scale would occur differently between core and non‐core taxa on shorter time scales (e.g., months).

We developed three specific hypotheses to investigate the core microbiome and general gut microbial diversity in North American red squirrels. The first hypothesis (H1) proposes that core microbial taxa will have few fluctuations in abundance because they are important for the organisation and diversity of the microbial community (Risely, 2020). The members of the core microbiome should thus be present in all gut microbial communities associated with the red squirrels, regardless of the host location, neighbouring host, or host environmental condition. Therefore, we predicted that (P1) no spatial patterns should prevail in the abundance of the core gut microbial taxa within the squirrel population. The second hypothesis (H2) proposes that the distribution of non‐core members of the gut microbiome will vary because of local interactions. The non‐core gut microbial taxa in red squirrel microbiomes are predicted to be spatially distributed (P2) due to local homogenisation of the red squirrel gut microbiome. Our first two hypotheses test if spatial patterns can be detected within core or non‐core gut microbiomes.

Third, we hypothesised that gut microbial community composition has a temporal component (e.g., varying across several months). Host behaviour and movement change seasonally, such as the territorial behaviours of red squirrels (Dantzer et al., 2012; Siracusa et al., 2019). Red squirrels allocate less time to territory defence in favour of mating opportunities and foraging fresh spruce buds in the early spring (Studd et al., 2020). Based on this increased host movement within squirrels' territories, we predicted that (P3A) there will be more indirect microbial transmission causing stronger small‐scale spatial patterns later in the season. In addition, these changes in host behaviour and movement will also change the identity of gut microbial taxa over time. Therefore, we predicted that there would be more differences in red squirrel gut microbiomes sampled at longer time intervals (i.e., more days apart), both within (P3B) and between (P3C) red squirrel individuals. Our final prediction on the importance of time incorporates the effect of local environmental conditions on host behaviour and movement. This prediction assumes that the same study area location will change less through time and thus predicts (P3D) that the differences in gut microbial community composition of the microbiome samples collected at the same location at increasing time periods will be smaller than the differences between samples collected at different locations.

## EXPERIMENTAL PROCEDURES

Ren et al. (2017) collected the North American red squirrel faecal samples analysed in this study from six 40‐hectare study areas near the Kluane National Park (Yukon, Canada) between 2008 and 2010. For all samples available for analysis (909), the sampling location was recorded as a within‐study area X‐Y coordinate (21–218 samples per year between February and August). DNA extracted from the samples was sequenced using the Illumina MiSeq platform (V1‐V3 16S rRNA). The 300 base pair sequence reads were filtered for quality using TRIMMOMATIC, with those passing the filtering step being merged using FLASH (Bolger et al., 2014; Magoč & Salzberg, 2011). Ren et al. (2017) discussed the previous analyses of this dataset in more detail.

Our study not only investigated different hypotheses compared to Ren et al. (2017), but we also used a modified bioinformatics approach. We first incorporated a compositional approach to replace read count‐based analyses, such as rarefaction and the Jaccard distance (Gloor et al., 2017; McMurdie & Holmes, 2014). Compositional analyses are better suited to microbiome studies, as the proportional read counts per sample are constrained to a constant sum (Gloor et al., 2017). Second, we expanded the previous spatial–temporal analyses by comparing core/non‐core gut microbiomes, and by analysing the data on a time scale of months rather than entire seasons. By grouping the data by month, we attempted to balance grouping with sufficient data (minimum six samples) with a short time scale.

Third, we opted to denoise the (post‐filtering and merging) DNA sequences as amplicon sequence variants (ASV) in QIIME2 (Callahan et al., 2016), rather than using the available 97% identity operational taxonomic units (OTUs) (Ren et al., 2017). Since the publication of Ren et al. (2017), there have been increased discussions of 97% identity OTUs being too low of a threshold for studies requiring higher taxonomic precision (Edgar, 2018; Johnson et al., 2019). This higher taxonomic resolution improved our analysis of non‐core taxa, many of which were less frequent in the data. We filtered the ASV data to retain only the ASVs that occurred in at least 1% of all samples (10 samples) to account for the possibility of sequencing errors (Dunphy et al., 2019; Edgar, 2018). After filtering, we assigned taxonomic labels to each ASV using a pre‐trained SILVA 138 reference database in QIIME2 (Quast et al., 2013; Yilmaz et al., 2014). We did not prioritise these taxonomic labels any further to prevent the loss of information about the microbial communities in downstream diversity analyses (Goodrich et al., 2014; Schloss & Westcott, 2015).

Our fourth key methodological difference was that we used a temporal abundance‐occupancy model method to identify the most important common ASVs in the red squirrel gut microbiome. Ren et al. (2017) previously defined the core gut microbiome on abundance alone. To determine the membership of the core gut microbiome, we ranked all ASVs based on their occupancy in microbial communities by collection dates, weighted by abundance, using code modified from Shade and Stopnisek (2019). The highest‐ranked ASVs in the occupancy‐abundance model samples were selected as core microbiome candidates (Table S4). Based on the core microbiome size and occupancy thresholds reviewed by Risely (2020), we selected the highest occupancy ASVs (>75%) identified by the occupancy‐abundance model to be members of the core microbiome. We did not identify a set of core ASVs specifically for each sampling study area to ensure that the defined core gut microbiome was generalisable to all samples.

In multivariate analyses, taking a compositional approach has been shown to have increased discriminatory power when analysing sparse count data like microbiome data (Martino et al., 2019) compared to the previous approach taken to analyse these data (Ren et al., 2017). We Aitchison‐transformed the ASV data from QIIME2 (*qiime2R* v0.99.13, *phyloseq* v1.28.0) prior to the principal coordinates of neighbour matrices (PCNM) analysis to account for the data's compositionality (Gloor et al., 2017; Quinn et al., 2018). The Aitchison transformation (*zCompositions* v1.4.0, *CoDaSeq* v0.99.6) involves the application of centred log‐ratios (clr) to imputed ASV count data (Aitchison, 1986; Gloor et al., 2017). We replicated this transformation and the spatial analysis for the core and non‐core ASVs found in each of the six study areas, for each month and year it was sampled to obtain our response matrix. We analysed the data per study area and per month to detect local transmissions. Grouping the samples in R for these analyses resulted in 29 study area‐month combinations.

The primary spatial multivariate approach used to construct spatial predictor variables was the principal coordinates of neighbour matrices (PCNM, *vegan* v2.6‐2). PCNM analysis identifies spatial structures in community composition data, focusing on neighbouring communities (Borcard et al., 2004; Borcard & Legendre, 2002; Buttigieg & Ramette, 2014; Dray et al., 2006). The nearest neighbours to the focal sample's location are weighted differently than those farther away in this principal coordinate analysis. Higher principle coordinate values in PCNM results correspond to finer‐scale spatial patterns (Borcard & Legendre, 2002). This multivariate approach has improved power relative to the distance decay analysis previously used to analyse these data (Ren et al., 2017).

The resulting PCNM data replicates were subsequently used for redundancy analysis (RDA) in R (v. 3.6.0; R Core Team, 2019). Forward selection was used to construct up to three significant RDA models for both the core and non‐core microbiome (predictions 1 and 2). These models were built stepwise using the adjusted *R* ^2^ values as a stopping criterion (Blanchet et al., 2008). We used analysis of variance (ANOVA) to calculate the significance of all RDA models through a permutation procedure (Anderson & Ter Braak, 2003). In addition, the adjusted *R* ^2^ values for the spatial models were fitted with a linear regression model against the interaction of month and community type (core or non‐core) to determine whether the variation explained by the spatial patterns varied through time (P3A). We used an ANOVA (*stats* v.3.6.0) to test the significance of the linear model.

We performed local regression (LOESS) in R (*stats* v.3.6.0) to further assess the influence of sampling location over time in different host individuals on core and non‐core microbial taxa. We first calculated the Aitchison distances between different host squirrels (*zCompositions* 1.4.0). These dissimilarity values were placed into one of the four groups, based on two sets of criteria: samples collected at the same location (same X‐Y coordinates) or a different location, and samples originating from the same host individual or a different individual (P3B, P3C, P3D). For the different squirrels, and different location groups, a random sample (10%) of the total sample comparisons were retained due to the high number of comparisons (see Table S3). Only comparisons between samples collected within the same study area‐month combinations in the same calendar year were analysed. We plotted the LOESS curves in R against the number of days between sample collection (*tidyverse* v.1.3.0). No specific statistical test was performed, as the shape of the relationship (the LOESS curves) was unknown.

## RESULTS

**FIGURE 1:**
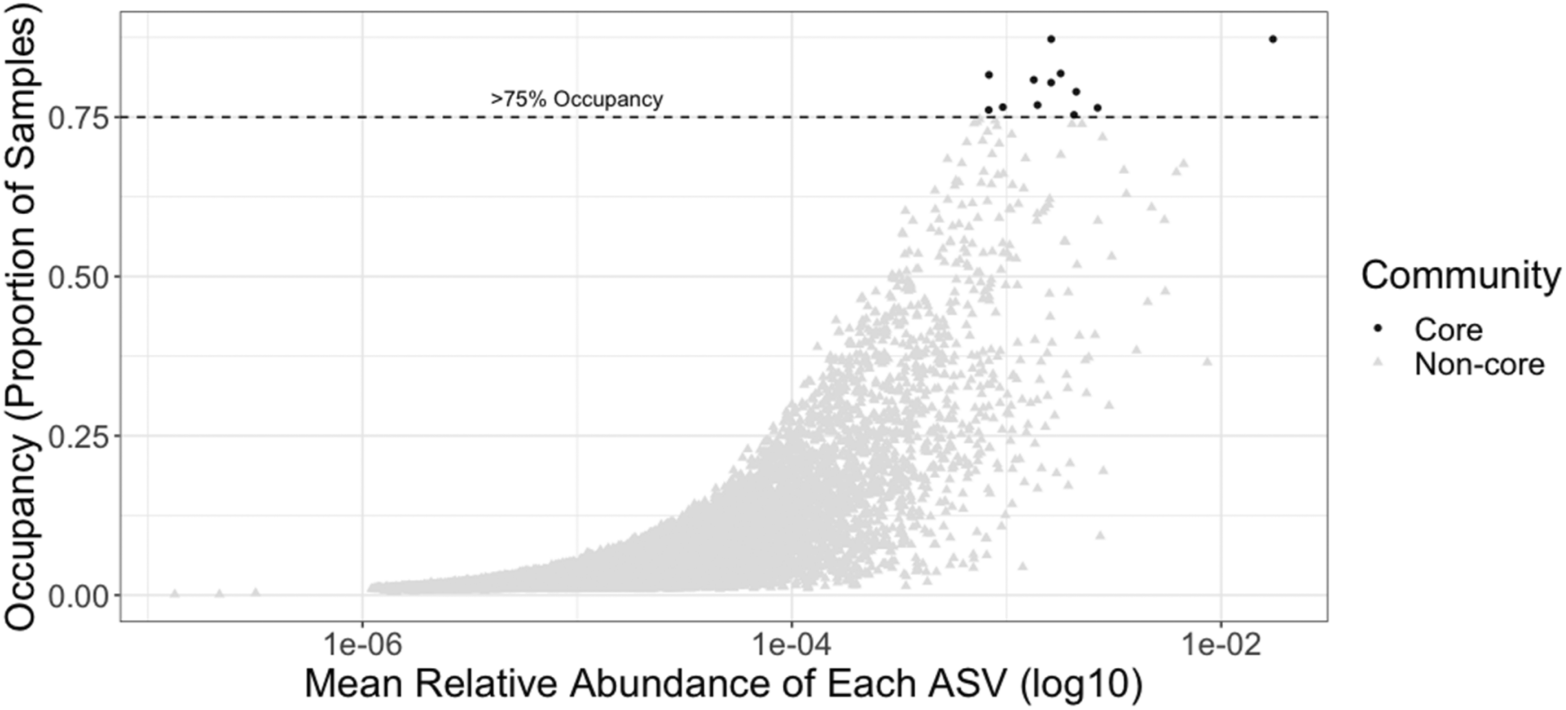
Scatterplot depicting the occupancy‐abundance distribution ASVs in red squirrel microbiomes. Points are shaped by whether the ASVs are members of the core or non‐core microbiome (75% occupancy threshold). ASV, amplicon sequence variant.

**FIGURE 2:**
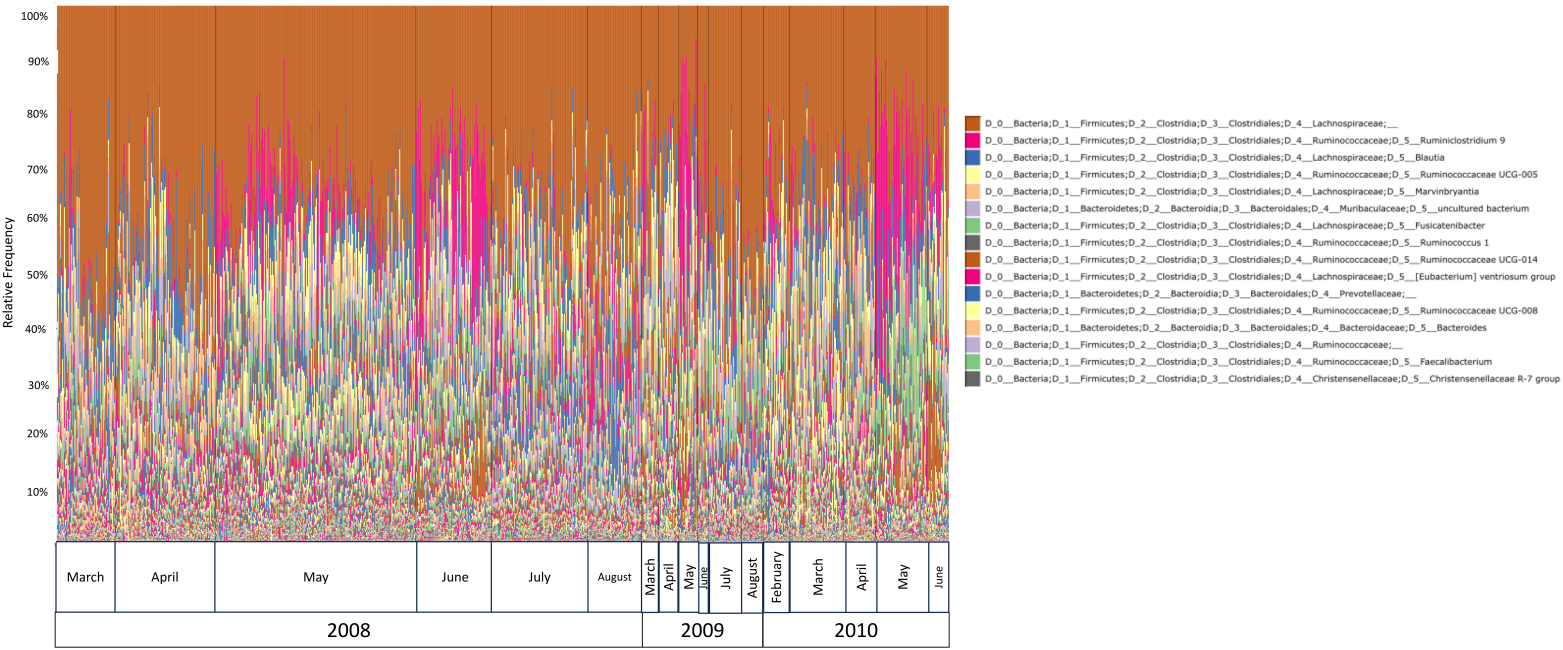
Taxonomic bar plot by month/year. Figure produced in QIIME2. Legend refers to the 16 most abundant genera. Full taxonomic identifications are available in Table S4.

We identified 12 core ASVs using an occupancy‐abundance method and a 75% occupancy threshold (Figure 1). The occupancy‐abundance model identified 141 ASVs as core, including 34 ASVs occurring in fewer than 50% of samples (Table S4). Knowing the core microbiome should be of high biological importance for the host, we were not confident that microbiota occurring in fewer than 50% of samples would have any more biological relevance than non‐core microbiota. We therefore selected an additional core occupancy threshold based on a discussion by Risely (2020) (70% threshold for a temporal core and up to 95% threshold for common core). The genera identified within the final core microbiome included: *Ruminiclostridium 9, Erysipelotrichaceae UCG‐003, Marvinbryantia, Ruminiclostridium 5, Marvinbryantia, Erysipelotrichaceae UCG‐003, Blautia*, two unlabelled *Lachnospiraceae* genera, *Ruminococcaceae UCG‐008*, one unlabelled *Prevotellaceae* genus and an ASV without taxonomic identification. While these core taxa were consistently present throughout the studied squirrel population (Figure 2), the number of ASVs present in the non‐core microbiome varied widely between hosts (12,303 to 37,992).

**TABLE 1:** Summary of the number of study area/month combinations with significant spatial patterns in the core and non‐core microbiome.

Season	Number of study area/month combinations	Number of study area/month combinations with significant spatial patterns	
		Core	Non‐core
Early Spring (February to April)	11	5 (45.5%)	8 (72.7%)
Late Spring (May and June)	12	4 (25%)	7 (58.3%)
Summer (July and August)	6	2 (33%)	4 (66%)
All Seasons	29	11 (37.9%)	19 (65.5%)

### Hypothesis 1: Limited spatial variation in core microbiomes

We identified over 100 significant spatial patterns (Table S1) for the 29‐study area‐month combinations of the core microbial community using PCNM analysis (P1, Table 1). Approximately one third (37.9%) of the study areas‐month combinations sampled across all months (February to August) had significant spatial patterns within the core microbial community (refuting P1). Within individual seasons, no more than half (45.5%) of the core communities had significant spatial patterns. These few (<50%) spatial patterns suggest that there are seasonal differences in the read count distribution of core microbial taxa within the overarching core definition. In comparison, significant spatial patterns were observed in a majority (>50%) of the non‐core gut microbial communities across all seasons (Table 1) as we predicted in Hypothesis 2.

### Hypothesis 2: Non‐core microbiomes vary spatially

**FIGURE 3:**
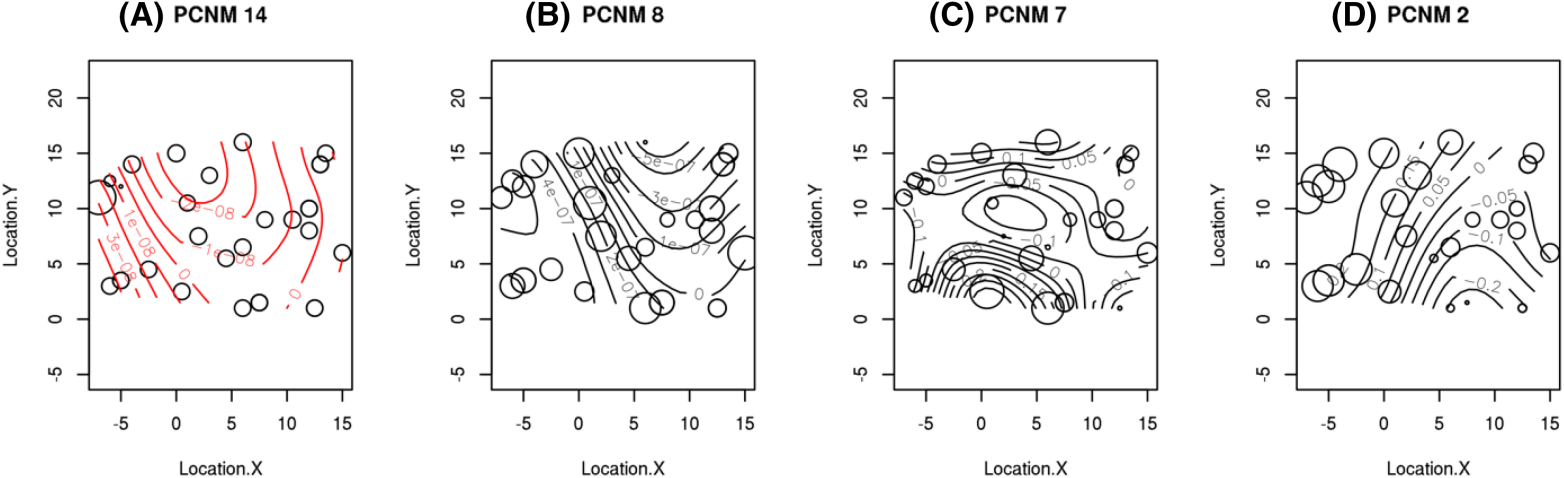
An example of significant spatial patterns from May 2008 in the Agnes (AG) study area. (A) (red)—core microbiomes; (B–D) (black)—non‐core microbiomes. Each unit distance corresponds to a 30‐metre interval staked in the study area. The circle sizes are proportional to the samples' PCNM score, while the generalised additive model (GAM) contour lines show non‐linear changes in microbiome composition.

We identified a series of putative spatial patterns in the distribution of ASVs across North American Red Squirrel fecal microbial communities. The non‐core gut microbial communities had a higher proportion of significant spatial patterns (Figure 3B–D) than the core gut microbial communities (Figure 3A), regardless of sampling month/season (Table 1). Within the core gut microbiome, the significant PCNM axes were approximately evenly divided between medium‐scale (PCNM number 11–20; 42%) and large‐scale (PCNM number 1–10; 46%) spatial patterns (Table S1). In contrast, most of the significant spatial patterns identified in the non‐core gut microbial communities were large‐scale (PCNM number 1–10; 67%). The large spatial patterns occur in samples collected the furthest apart in a study area (up to 930.1 m apart; Table S2). Considering these within‐study area spatial patterns, the results suggest a localised (i.e., <930.1 m, within a study area) distribution in gut microbial community composition.

### Hypothesis 3: Monthly variation in microbiome composition

**FIGURE 4:**
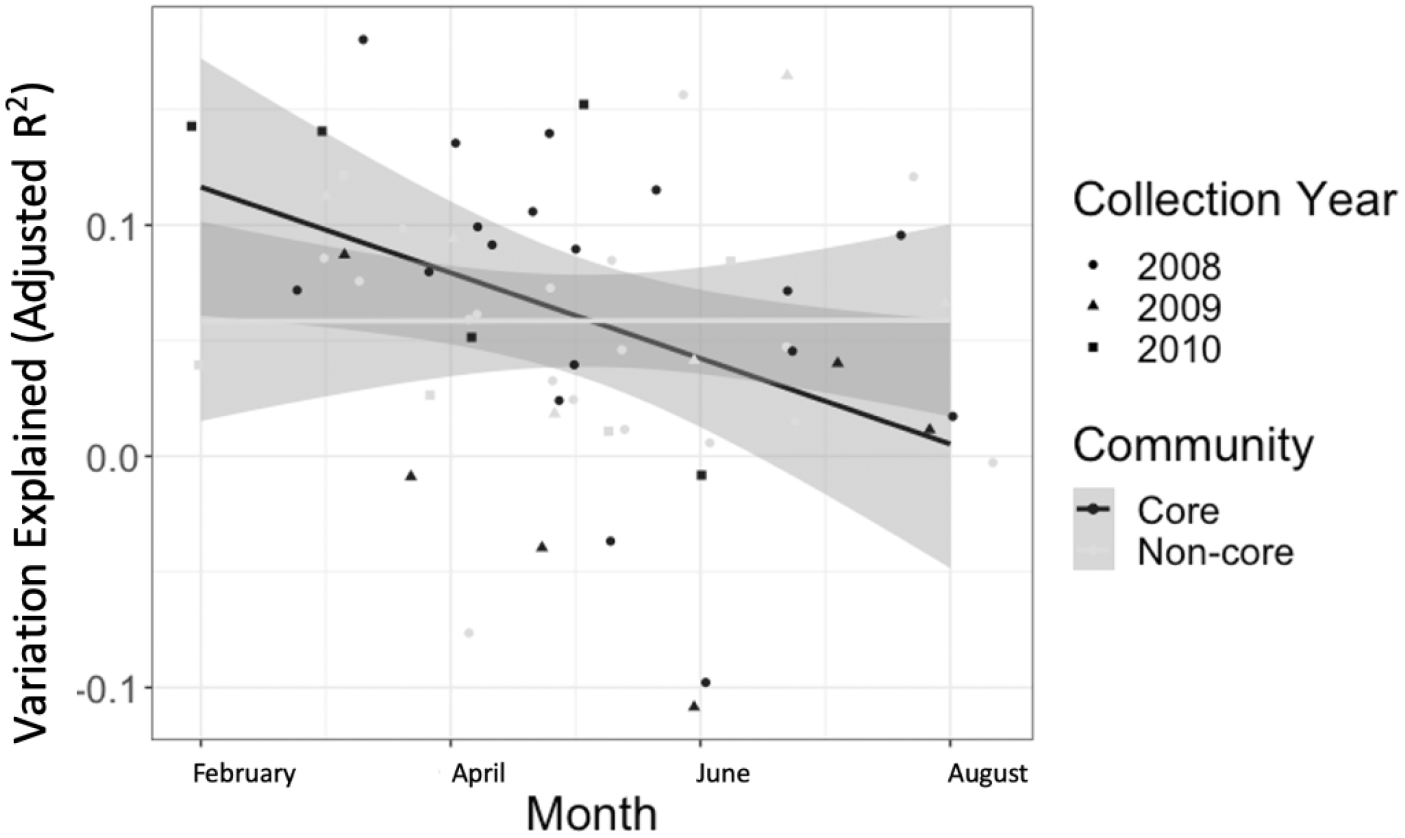
Variation explained (adjusted *R*‐squared values) by spatial patterns from the RDA models by month in the core and non‐core microbial communities. Each point corresponds to a study area where sufficient samples were collected in that month. Jitter has been introduced to the points to ensure points visibility. The grey shading around each regression line represents one standard error from the linear model line.

We hypothesised that there would be seasonal variations in the distribution of squirrel gut microbiomes (H3). During the sampling period from spring to summer, we observed a significant difference in the variation explained (adjusted *R* ^2^ values) by the spatial patterns in the core gut microbiome (ANOVA, *p* = 0.026, P3A). Furthermore, the adjusted R^2^ values varied greatly between study area‐month combinations, particularly in the core gut microbiomes (Figure 4, CV_core_ = −0.018512, CV_non‐core_ = 0.0000654). These results suggest that the core gut microbial taxa occur under different environmental selection pressures depending on the sampling month at each host location.

**FIGURE 5:**
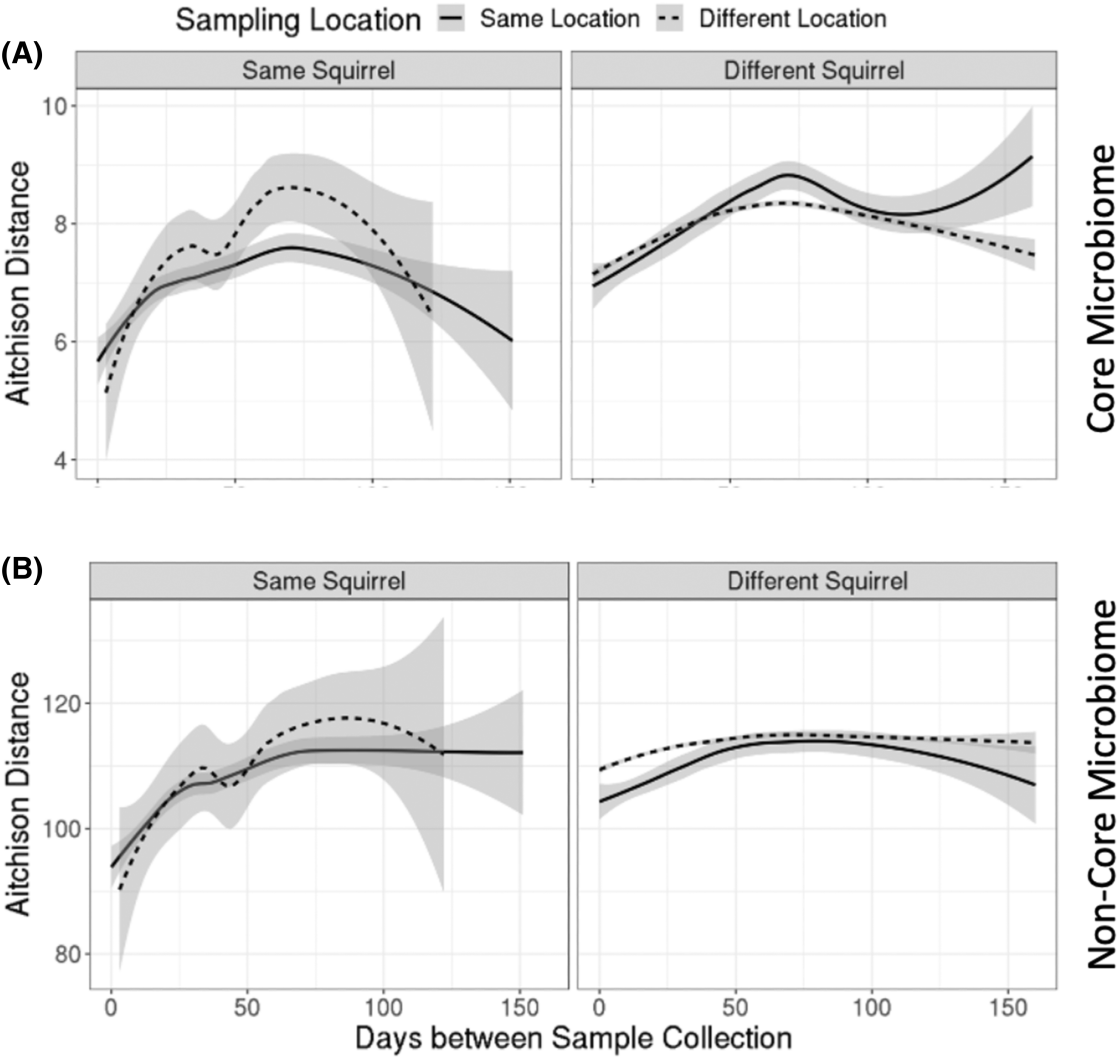
Local regression (LOESS) curves of Aitchison distance (dissimilarity) between microbiome samples over time (*n*_comparisons_ = 122 to 15,992). (A) Core microbiomes; (B) Non‐core microbiomes. The grey shading around each regression line represents one standard error from the curve. Y axes are different for core and non‐core comparisons.

We used non‐parametric local regression to show that the dissimilarity between microbial communities varies spatiotemporally (Figure 5). As expected, the dissimilarity between non‐core gut microbial communities was far higher than between core communities, regardless of the time elapsed between samplings. For squirrels whose gut microbiomes were resampled, we observed an increasing dissimilarity between gut microbial communities regardless of the location at which the samples were collected (P3B, Figure 5—left panels). This observation suggests that there are seasonal changes in microbial composition, regardless of individual sampling location or which microbial taxa are being considered. When comparing the microbiome of different squirrels (P3D), similar rates of change in community dissimilarity were observed over time regardless of the sampling location (Figure 5A,B—right panels). The non‐core microbiome (Figure 5B—right panel) showed lower dissimilarity when the samples were collected at the same location (P3D). We also observed a sudden uptick in core microbiome dissimilarity for squirrels in the same location at the end of the sampling period (Figure 5A—right panel).

## DISCUSSION

We developed three hypotheses regarding the factors influencing the small‐scale distribution of microbial taxa in the gut microbiomes of North American red squirrels. The 12 ASVs in the core gut microbiome were predicted to be differentially influenced by external factors (e.g., host location) compared to the non‐core gut microbiome. However, replicate tests on this large data set suggest the external factors do not differentially impact core and non‐core ASVs. We observed spatial patterns in the distribution of core ASVs in 41% of the study area‐month combination, where we only expected spatial patterns in the non‐core ASVs. In addition, we observed stronger seasonal changes in variation explained by spatial patterns in the core gut microbiome than in the non‐core gut microbiome (Figure 4).

We predicted that the core microbial taxa would not vary spatially (P1), as these taxa were expected to carry out essential functions for host and microbial community health (Lemanceau et al., 2017; Risely, 2020). However, we observed significant spatial patterns in the distribution of the core microbial taxa, though 20% fewer spatial patterns than in the non‐core microbial taxa (Table S1). These spatial patterns suggest the possibility of local homogenisation (i.e., within study areas, up to 930.1 m between sampling locations), even within members of the core gut microbiome. The host location may have had a greater influence on core microbial taxa than expected in our study, possibly due to the environments of neighbouring North American red squirrels. While localised spatial patterns have previously been observed in free‐living core microbiomes (Jiao et al., 2019), we expected the importance of host association to minimise the influence of host location.

The unexpected spatial patterns in the distribution of core gut microbial taxa across months (Figure 4) may have been a result of seasonal physiological variations between individual squirrels (Larivée et al., 2010). For example, there have been associations between squirrel glucocorticoid levels and microbiome composition (Petrullo et al., 2022; Stothart et al., 2016). Glucocorticoids have also been studied in association with microbiome composition in other systems across different populations and seasons (Dantzer et al., 2016; Kothmann et al., 2022; Rudolph et al., 2022). Furthermore, individual host traits may result in differential rates of microbial survival as well as context‐specific functions after microbial transmission to a given host (Robinson et al., 2019). To make conclusions about the relationships between the microbiome relationship to host behaviour (Cryan & O'Mahony, 2011), further studies on North American red squirrels are needed. For example, an RNAseq study combined with behavioural data would provide better conclusions about microbial functions in the Kluane population.

In contrast to the core gut microbiome, we predicted that the non‐core gut microbiome would exhibit a variety of spatial patterns (P2). We observed that most sampling study areas had significant spatial patterns, regardless of the sampling month (Table S1). The consistent frequency in spatial patterns across months and study areas suggests highly localised distributions in the red squirrel microbiome. These localisations are probably the result of shared selective pressure events between neighbouring hosts. For example, during the early spring season (February to April), the spatial patterns would coincide with the potentially homogenising effect of reliance on stored foods before fresh food becomes available in the spring (Ren et al., 2017). Later in the spring and summer (May to August), an alternative process explaining the consistent frequency in spatial patterns could be exposure to similar environmental factors (Lemanceau et al., 2017). Furthermore, early‐life diets may be a homogenising factor in red squirrel faecal microbiomes.

Increased variation in community composition was predicted to be explained by a host's location in their environment (Figure 3A–D). We also expected the host location to drive an increase in variation between red squirrel gut microbiomes. Seasonal changes in territory defence (Vlasman & Fryxell, 2002) and other host behaviours may increase microbial transmission events in the spring and summer (Robinson et al., 2019). These behavioural changes could explain the rapid change in microbial communities at short time intervals (Figure 5). Despite similar dispersal, the faecal microbiomes of mother‐infant pairs in this population remained similar (Ren et al., 2017). Vertical transmission may therefore result in more persistent microbiome signatures than horizontal transmission across already‐populated microbiomes (Valles‐Colomer et al., 2023). Regardless of the means of microbial dispersal, there is little change in variation explained by spatial patterns in the non‐core gut microbiome as time progresses (Figure 4). This lack of change suggests that host location consistently contributes to microbial community assembly in non‐core gut microbiomes because environmental transmission overshadows any horizontal transmission between individuals.

Direct observations of the rates with which North American Red Squirrel interact during sampling would provide a test of the frequency of horizontal transmission. Some estimates for these infrequent interactions have previously been collected for the Kluane Red Squirrel population (Dantzer et al., 2012). In addition, combining temporal sampling of the faecal microbiome before and after interactions between two individuals would provide a detailed test of the importance of these specific behavioural interactions to gut microbiome similarity. Reassessing the state of faecal microbiomes from the Kluane red squirrel population in a few years could further indicate the spatial stability in microbial metacommunities over longer periods.

Another contrast with Ren et al. (2017) was that they found evidence for oscillations of two core genera (*Coprococcus* in early spring and summer, and *Oscillospira* in late spring). Neither of these genera previously identified as core gut microbiota in this population met our updated criteria to be considered part of the core microbiome (Ren et al., 2017). In our updated analysis, only the core microbial taxa showed potential evidence for oscillations with microbiomes after 75–150 days becoming more similar again (see Figure 5A). If those two genera identified by Ren et al. (2017) were important for the microbiome turnover, we expect to have found evidence for oscillation in the non‐core analyses, the opposite of our results. Further sampling will be required to investigate these potential cyclical patterns in this dynamic microbiome/host system.

Defining what constitutes the core microbiome has presented a challenge because of the lack of standardisation in the literature (Risely, 2020). This lack of standardisation is supported by our contrasting finding in core gut microbiome oscillations, which may be an artefact of using a different bioinformatic approach to define the ‘core’ microbiome than Ren et al. (2017). Furthermore, we observed no apparent difference in the occupancy‐abundance distribution of ASVs that fell above the 75% occupancy threshold (Figure 2). Lowering the occupancy threshold may have revealed additional spatial patterns within the core microbiome composition. However, the core ASVs are important for microbial community stability because of their widespread nature. Our findings reiterate a need for further discourse on the biological importance of core microbial taxa and how these taxa are identified in microbial ecology research (Neu et al., 2021; Risely, 2020).

## CONCLUSIONS

We demonstrated that the spatial–temporal distribution of host individuals can have a significant impact on the non‐core, but also surprisingly, on the core microbial taxa present in faecal microbiomes. Moreover, this pattern was repeatable over several locations and years; such replication is not seen in many microbiome studies. This replication was only possible through the unique, long‐term study of hosts and their associated microbiomes. In addition, sampling exclusively within one season and/or location could lead to incorrect conclusions. For example, replication produced through the monthly time scale we used to analyse the data across different locations has strengthened our conclusions. Developing microbiome studies like “traditional” (macro)ecology research (i.e., incorporating multiple locations, years/times) will increase the reliability of the conclusions drawn from highly variable microbiome samples and further inform laboratory‐based microbiome research.

## AUTHOR CONTRIBUTIONS

**Alicia Halhed:** Data curation (lead); formal analysis (lead); visualization (lead); writing – original draft (lead); writing – review and editing (lead). **Lauren Petrullo:** Writing – review and editing (supporting). **Stan Boutin:** Funding acquisition (equal); investigation (equal); methodology (equal); project administration (equal); resources (equal); writing – review and editing (supporting). **Ben Dantzer:** Funding acquisition (equal); investigation (equal); methodology (equal); project administration (equal); resources (equal); writing – review and editing (supporting). **Andrew McAdam:** Funding acquisition (equal); investigation (equal); methodology (equal); project administration (equal); resources (equal); writing – review and editing (supporting). **Martin Wu:** Funding acquisition (equal); investigation (equal); methodology (equal); project administration (equal); resources (equal); writing – review and editing (supporting). **Karl Cottenie:** Conceptualization (lead); methodology (equal); supervision (lead); validation (equal); writing – review and editing (equal).

## CONFLICT OF INTEREST STATEMENT

The authors have no conflicts of interest to declare.

## ETHICS STATEMENT

In completing this research, we have abided by all relevant regulations, conventions and ethical practices.

## Supporting information


**TABLE S1:** Significant PCNM axis numbers by sampling grid, month, and year. Grids marked ‘yes’ in the food column received peanut butter as food.
**TABLE S2:** Total number of 99% identity ASVs in each grid/year combination. The number of samples, the percentage of the sample size that were individuals.
**TABLE S3:** Number of microbiome sample comparisons available for plotting in Figure 4.
**TABLE S4:** Taxonomic assignments and groupings (core vs. non‐core) for each ASV.

## Data Availability

All associated script files used in these analyses are available in a dedicated GitHub repository (https://github.com/ahalhed/Red-Squirrel-Microbiome). The ASV table and other resulting data files are available upon request. The sequence and sample data files were previously made publicly available in the figshare repository associated with Ren et al. (2017): https://figshare.com/s/a52886d8016cdd1f0dbb.
